# Genetic effects of ATP1A2 in familial hemiplegic migraine type II and animal models

**DOI:** 10.1186/1479-7364-7-8

**Published:** 2013-04-05

**Authors:** Stephanie M Gritz, Richard A Radcliffe

**Affiliations:** 1 Department of Pharmaceutical Sciences, University of Colorado Anschutz Medical Campus, Aurora, CO, 80045, USA; 2 Institute for Behavioral Genetics, University of Colorado, Boulder, CO, USA

**Keywords:** Atp1a2, Ions, FHM2, Migraine, Knock-out mouse, Anxiety, Fear, Learning, Memory

## Abstract

Na^+^/K^+^-ATPase alpha 2 (Atp1a2) is an integral plasma membrane protein belonging to the P-type ATPase family that is responsible for maintaining the sodium (Na^+^) and potassium (K^+^) gradients across cellular membranes with hydrolysis of ATP. Atp1a2 contains two subunits, alpha and beta, with each having various isoforms and differential tissue distribution. In humans, mutations in *ATP1A2* are associated with a rare form of hereditary migraines with aura known as familial hemiplegic migraine type II. Genetic studies in mice have revealed other neurological effects of *Atp1a2* in mice including anxiety, fear, and learning and motor function disorders. This paper reviews the recent findings in the literature concerning *Atp1a2*.

## Introduction

**Figure 1:**
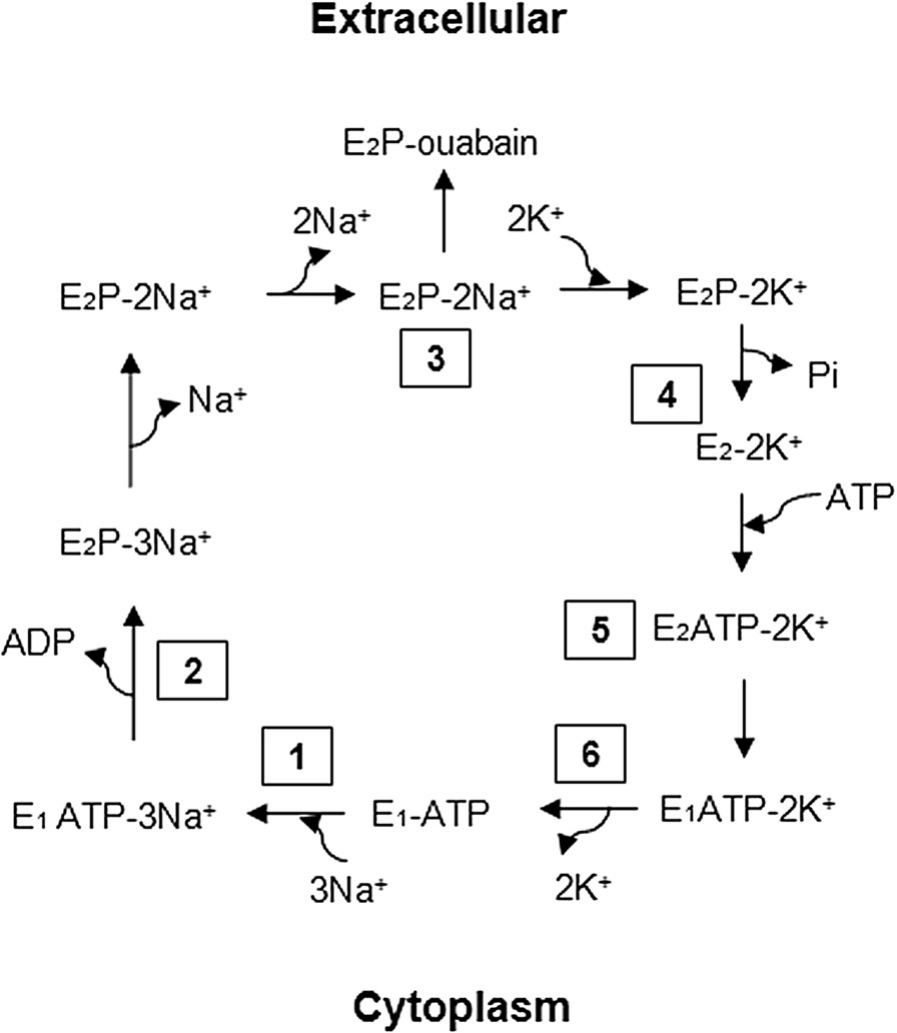
**The Na**^**+**^**/K**^**+**^**-ATPase reaction cycle.** The sequence of steps involved in the active transport of Na+ and K+ ions. See text for details [1-3].

The integral plasma membrane protein Na^+^/K^+^-ATPase is a member of the P-type ATPase family. P-type ATPases maintain the essential plasma membrane potential in all eukaryotic cells and are found in all cell type membranes. The plasma membrane potential is upheld by adjusting ion concentrations on the intracellular and extracellular sides of the membrane. This electrochemical gradient fuels central cellular processes, such as the secondary transport of metabolites, and it also provides the basis for electrical excitation in neurons. The mechanism of action of Na^+^/K^+^-ATPase involves a conformation change driven by ATP hydrolysis (Figure 1). While bound to ATP, the pump binds three intracellular sodium ions (Figure 1, step 1). ATP is then hydrolyzed to ADP, and the pump is phosphorylated at a highly conserved aspartate residue (Figure 1, step 2). This phosphorylation causes a conformation change, resulting in the release of the sodium ions into the extracellular space and the binding of two potassium ions (Figure 1, step 3). This binding of potassium ions causes the pump to dephosphorylate (Figure 1, step 4), which returns the pump to its previous conformational state (Figure 1, step 5), and transports the potassium ions into the cell (Figure 1, step 6). The process then repeats itself when ATP again binds to the pump.

The Na^+^/K^+^-ATPases consist of an α subunit and a β subunit and, only in the kidney, a γ subunit [1,4]. The α and β subunits are synthesized separately, assembled in the endoplasmic reticulum, and are delivered to the plasma membrane [1,5]. The α subunit is the catalytic region of the enzyme and contains the binding sites for the sodium and potassium ions, ATP, and cardiac glycosides such as ouabain, while the glycosylated β subunit is needed for proper folding and function of the catalytic subunit. The X-ray crystal structure of Na^+^/K^+^-ATPase was examined from pig kidney, which showed the binding sites in the α subunit and the interactions between the α and β subunits [6]. Different genes encode each of the multiple α and β isoforms and the γ isoform; four α isoforms (*ATP1A1*, *ATP1A2*, *ATP1A3*, and *ATP1A4*), four β isoforms (*ATP1B1*, *ATP1B2*, *ATP1B3* and *ATP1B4*), and one γ isoform (*FXYD2*) have been identified (Table 1, [7,8]).

**Table 1:** **Na**^**+**^**/K**^**+**^-**ATPases in the P-type ATPase family in the human and mouse**

**Type**	**Human**	**Location (chr:bp)**	**Size (#AA)**	**Mouse**	**Location (chr:bp)**	**Size (#AA)**	**Homology (%)**
α1	ATP1A1	1:116915795	1023	Atp1a1	3:101576219	1023	97
α2	ATP1A2	1:160085520	1020	Atp1a2	1:172271709	1020	99
α3	ATP1A3	19:42470734	1013	Atp1a3	7:24978167	1013	99
α4	ATP1A4	1:160121352	1029	Atp1a4	1:172223508	1032	83
β1	ATP1B1	1:169075947	303	Atp1b1	1:164437267	304	94
β2	ATP1B2	17:7554254	290	Atp1b2	11:69599750	290	97
β3	ATP1B3	3:141595470	279	Atp1b3	9:96332673	278	73
β4	ATP1B4	X:119495940	357	Atp1b4	X:38316267	356	89
γ	FXYD2	11:117690790	64	Fxyd2	9:45399709	64	81

The α isoforms differ in tissue distribution and are regulated developmentally [9]. The α1 subunit is expressed ubiquitously, the α2 isoform is expressed in the brain, heart, and skeletal muscle, the α3 subunit is expressed in the brain and heart, and the α4 isoform is expressed in sperm and its precursor cells [10,11]. The four isoforms have a high degree of amino acid identity but have differences in kinetic properties and substrate affinity [12,13]. Homology between human and mouse protein is between 83% and 99%, and within subunits and species, it is approximately between 80% and 90% (Table 1) [14]. In the adult brain, the α1 isoform is found in multiple central nervous system (CNS) cell types, the α2 isoform is primarily expressed in astrocytes and pyramidal cells in the hippocampus, and α3 is expressed only in neurons [15-17].

ATP1A2, as well as the other isoforms, is responsible for maintaining the resting membrane potential and for driving nutrient and neurotransmitter uptakes. Na^+^/K^+^-ATPases are important in clearing extracellular potassium during neuronal activity and are essential in the clearance of released glutamate in the synaptic cleft because reuptake in astrocytes and neurons is driven by the sodium and potassium gradients [18]. ATP1A2 is co-localized with other ion transporters, such as the sodium/calcium (Na^+^/Ca^2+^) exchanger and the glutamate transporter, which are important in the clearance of glutamate and potassium from the extracellular space in the CNS [19-21].

### The role of *ATP1A2* in FHM2

Familial hemiplegic migraine (FHM) is a rare autosomal dominant form of migraine with aura. FHM attacks are generally longer than the common migraine with aura; however, they share similar symptoms (Table 2, [22]) including visual, sensory, motor, and aphasia [23]. Aura symptoms are different for each person but are generally described as disturbances in light perception, such as spots or lines in vision, and changes in sensory perception, such as heightened sensitivity to smells and sounds [24]. There are three types of FHM: type 1 is associated with mutations in the neuronal calcium channel gene and is the most prevalent form of FHM, type 2 is caused by mutations present in *ATP1A2*, and type 3 is a rare form of FHM related to mutations in the sodium channel gene [24].

**Table 2:** International Headache Society migraine criterion

**Repeated episodic headache features within 4 to 72 h**	
Any two of the following	Any one of the following
Unilateral	Nausea and/or vomiting
Throbbing	Photophobia and phonophobia
Worsened by movement	
Moderate or severe	

FHM2 patients suffer from migraines with hemiplegia and partial paralysis during the aura phase and, in some cases, accompanied by seizures or cognitive dysfunction. Neuroimaging studies have shown that migraine aura is caused by cortical spreading depression (CSD). CSD is a wave of continual strong neuronal depolarization that slowly progresses across the cortex, generating a brief intense spike of activity that is followed by long-lasting neural suppression [25]. CSD has been shown to activate the trigeminovascular system (TGVS), which is responsible for the headache associated with migraines [24]. Inhibition of ATP1A2 leads to high levels of extracellular potassium, causing neurons to become depolarized which can cause CSD [26].

*ATP1A2* was identified as a gene associated with FHM2 in 2003 in two Italian families [27]. The mutations and deficiencies in *ATP1A2* that cause FHM2 are responsible for approximately 20% of FHM in families [2]. There are over 50 mutations in ATP1A2 that have been identified in association with FHM2. Almost all FHM2 mutations are non-synonymous SNPs, but there are also small deletions [28] and a mutation affecting the stop codon, causing an extension of the *ATP1A2* protein by 27 amino acid residues [29]. Most of the mutations are associated with pure FHM without additional clinical symptoms [27-31]. Recently, a number of *ATP1A2* mutations were reported to be associated with FHM and cerebellar problems, specifically motor problems [32], childhood convulsions [33], epilepsy [29,34], and mental retardation [29,35]. Some *ATP1A2* mutations have been shown to be associated with non-hemiplegic migraine phenotypes, such as basilar migraine [36] and the common migraine [37].

One of the most important experimental drugs to study ATP1A2, as well as ATP1A1 and ATP1A3, is ouabain which is a plant-derived steroid that binds to the E_2_P form of Na^+^/K^+^-ATPases, as seen in Figure 1. Ouabain acts as a non-selective antagonist and inhibits the enzyme transport activity [38]. The three isoforms in humans show similar affinity for ouabain; however, ouabain binding is altered due to changes in extracellular potassium levels, resulting in varying sensitivities [39]. In rats, Atp1a3 is very sensitive to ouabain, Atp1a2 is less sensitive, and Atp1a1 is insensitive [40].

Cell-based studies have shown that multiple individual mutations in *ATP1A2* result in dysfunctional ion pump activity. The functional effects of mutations in *ATP1A2* have been studied in HeLa cells and *Xenopus* oocytes (Table 3). Recombinant *ATP1A2* subunits containing mutations that cause the pump to be insensitive to ouabain were expressed in HeLa cells; the ouabain-insensitive mutants were used to distinguish between endogenous ATP1A2 activity and the effects of other mutations engineered into the recombinant ATP1A2 [27,33,35,41,42]. Three mutations in HeLa cells and two mutations in *Xenopus* oocytes produced severe or complete loss of function of pump activity, which led to cell death [3,27,35,41]. Five other FHM2 mutants were analyzed, and pump activity was reduced but sufficient to allow survival of the HeLa cells [41-43].

**Table 3:** **Cell-based studies demonstrating the effect of ATP1A2 mutations in *Xenopus *oocytes and HeLa cells**

**Cell type**	**Mutation**	**Effect on pump function**	**Reference**
HeLa	L764P	Complete loss	[2,3,27]
	W887R	Complete loss	[2,3,27]
	G615R	Complete loss	[27]
	R593W	Reduced rate	[2,41,42]
	V628M	Reduced rate	[2,41,42]
	T345A	Similar to wild-type: lower affinity for potassium	[2,41,42]
	M731T	Similar to wild-type: decreased catalytic turnover and increased affinity for potassium	[2,41,42]
	R689Q	Similar to wild-type: decreased catalytic turnover and increased affinity for potassium	[2,41,42]
*Xenopus* oocytes	L764P	Complete loss	[43]
	W887R	Complete loss	[43]

ATP1A2 is expressed primarily in astrocytes in the adult, where it appears functionally coupled to various transporters (glutamate transporter and Na^+^/Ca^2+^ exchanger), and is essential in the clearance of released glutamate and potassium from the extracellular space during neuronal activity. FHM2 mutations that cause loss of function of ATP1A2 may lead to decreased glutamate clearance and an increase of potassium in the synaptic cleft during neuronal activity, which could lead to prolonged recovery time after neuronal excitation, and may render the brain to be more susceptible to CSD [23,26].

There are two hypothesized mechanisms for the effects of ATP1A2 mutations. First, the mutations cause an increase in extracellular potassium, which can result in the impaired clearance of potassium ions and therefore induce CSD [43]. Second, since the distribution of ATP1A2 is co-localized with the Na^+^/Ca^2+^ exchanger, the mutations to ATP1A2 would cause intracellular sodium to increase, which increases intracellular calcium levels through the Na^+^/Ca^2+^ exchanger, similar to FHM1, resulting in glutamate release and a decrease in glutamate clearance which can also lead to CSD [23,44]. Both hypotheses result in making the brain more susceptible to CSD and therefore migraines with aura.

Currently, the medications used to treat FHM2 are standard migraine prophylactic drugs, such as antidepressants, beta blockers, and calcium channel blockers [45], which treat the symptoms, not the cause. Further investigation of ATP1A2 in humans and animal models are needed to better determine treatment options.

### Atp1a2 studies in animal models

**Figure 2:**
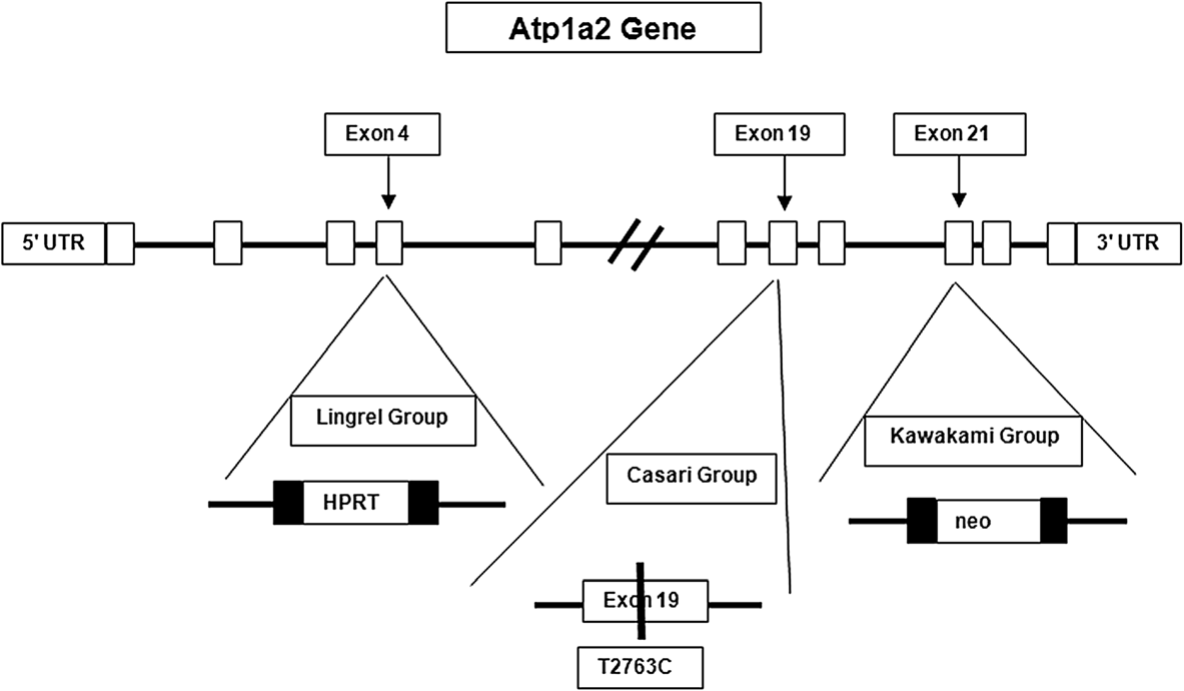
**Three separate groups have mutated the *Atp1a2 *gene in mice.** Two groups created knock-outs through disruption of the gene at two different locations and the other has created a knock-in mutation to mimic one of the FHM2 mutations in exon 19. *Atp1a2* is located in the antisense strand of chromosome 1 [18,19,26].

Over the last 20 years, animal studies using either *Atp1a2* knock-out or knock-in mutations have increased our understanding of its effect on behavior. *Atp1a2* heterozygous mice have been studied by two separate groups, one at the University of Cincinnati led by Dr. Jerry Lingrel and the second at Jichi Medical School in Japan overseen by Dr. Kiyoshi Kawakami. Both groups have shown that modulation of *Atp1a2* activity affects neural activity and whole animal behavior. More recently, a group led by Giorgio Casari at the Vita-Salute San Raffaele University and Center for Translational Genomics and Bioinformatics in Italy generated the first FHM2 knock-in mouse. The constructs for the gene alterations are shown in Figure 2.

Lingrel's lab has been investigating the functional roles of the various Na^+^/K^+^-ATPase isoforms for over 20 years. This lab developed heterozygous mice for *Atp1a2* as well as for *Atp1a1* and *Atp1a3*[19]. *Atp1a2* heterozygous (*Atp1a2±*) mice were tested for differences in behavior as well as to investigate the role of *Atp1a2* in the heart. Kawakami's group also created an *Atp1a2*± mouse by deleting a portion of the gene, resulting in only one functioning copy [18]. They examined the role of Atp1a2 in neural activity by measuring fear and anxiety in heterozygous mice. The *Atp1a2* heterozygous mice also showed hyperphagia during the light period and suffered from late onset obesity [46]. The homozygous null *Atp1a2* mice are neonatally lethal due to lack of synchronized neuronal firing in the breathing center of the brain [16,47,48], and the *Atp1a2±* mice have approximately half the protein compared to with wild type [19].

The heterozygous mice from both groups consistently show an anxiety-related phenotype (Table 4). The *Atp1a2±* mice were hypoactive compared with the wild type based on measurements of total distance traveled and time spent in the corners of the open-field testing box [17]. They also spent less time in and had fewer entries into the open arm of the plus maze [11,17,41]. In the light/dark test, the knock-outs spent less time in the light compartment and had fewer transitions between the two compartments [18]. Taken together, these results show that the *Atp1a2*± mice are more anxious than the wild type.

**Table 4:** Testing in the Atp1a2 heterozygous mice

**Test**	**Phenotype measured**	**Parameters measured**	**Heterozygous compared with wild type**	**Lab**
Hidden platform	Learning and memory	Latency time	Decreased	Lingrel
Elevated zero maze	Anxiety	Time spent in open arms	Decreased	Lingrel
		Entries into open quadrant	Decreased	
Open-field activity	Anxiety	Time spent in corners	Increased	Lingrel
		Total distance traveled	Decreased	Lingrel and Kawakami
Light/dark	Anxiety	Path length in light compartment	Decreased	Kawakami
		Time spent in light compartment	Decreased	
		Latency to first entry into light compartment	Increased	
		Number of transitions between light and dark	Decreased	
Elevated plus maze	Anxiety	Time in open arms	Decreased	Kawakami
		Entries into open arms	Decreased	
Conditioned fear stimuli	Fear and learning	Freezing time	Increased	Kawakami
Cardiac performance	Hypercontractile heart	Contractibility	Increased	Lingrel

The various tests, parameter measured, and results were compared with the wild type [17,18].

Learning and memory behavioral tests were interpreted differently between the two groups. The Lingrel lab examined spatial learning and memory using the Morris water maze and found that the knock-out mouse had a longer latency to find the hidden platform, suggesting that they have impaired learning. The Kawakami lab studied the knock-out mouse in the conditioned fear test, which showed that the knock-out mouse had increased freezing time which is typically interpreted as improved learning and memory [18]. However, the authors concluded that the knock-out mouse had enhanced fear; other effects such as increased learning and memory and altered sensory perception could explain the results. The authors did not examine or control these other possibilities.

The role of Atp1a2 has also been examined in the hearts of the heterozygous mice. While the hearts were histologically the same in *Atp1a2±* and wild-type mice, the *Atp1a2*± mice had hypercontractile hearts [19,41,42]. As noted above, the *Atp1a2*± mice have 50% less protein than the wild type, and reduction of Atp1a2 alters calcium levels in cardiomyocytes, suggesting that the intracellular concentration of sodium ions would increase. This increased concentration results in an increase in intracellular calcium levels because the sodium-calcium exchanger is inhibited by the high intracellular sodium levels. The excess intracellular calcium causes the contractions in the cardiomyocytes to increase in strength [19,41]. These results suggest that Atp1a2 regulates calcium levels and, therefore, contractibility in the heart.

The group at the Vita-Salute San Raffaele University and Center for Translational Genomics and Bioinformatics in Italy has generated the first FHM2 knock-in mouse. This mouse model was created by inserting the T2763C mutation, in exon 19 of the mouse, which is one of the first mutations that was associated with FHM2 [26,41,42]. The T2763C mutation causes the amino acid substitution W887R, which affects the β subunit binding site in *Atp1a2,* resulting in a misfolding of the protein and, in cell-based studies, a complete loss of pump function. The homozygous knock-in mice are neonatally lethal, similar to the knock-out mice described previously; therefore, heterozygous knock-in mice were used for experiments. The authors suggested that because the W887R mutant protein does not translocate efficiently to the plasma membrane, it is degraded via the proteasome, which results in reduced overall protein in the brain. The knock-in mice were tested for CSD by electrical stimulation, and results showed that they were more susceptible to CSD: they had a higher threshold and velocity but equal duration of CSD than the wild type. These results may be due to the impaired clearance of extracellular potassium and glutamate by astrocytes, which is comparable to the effects of FHM2 mutations in humans [23,26]. Further testing on this mouse model will be beneficial to understanding the mechanism of at least this mutation in FHM and possible treatment avenues.

## Conclusions

Mutations and deficiencies in *Atp1a2* are associated with FHM2 in patients suffering from migraines with hemiplegia and partial paralysis during the aura phase. FHM2 patients may also have increased susceptibility to CSD, which may be due to the increase in extracellular potassium and/or a decrease in glutamate clearance. CSD has been shown to cause the aura coupled with migraines and may activate the TGVS, resulting in the headache portion of a migraine.

Studies in *Atp1a2*-deficient mice suggest an association of the protein with anxiety, learning disorders, and fear. Multiple groups have created knock-out and knock-in heterozygous *Atp1a2* mice and have tested them for fear and anxiety, locomotor activity, learning ability, and susceptibility to CSD. The results of these animal studies show significant increased anxiety and fear, reduced locomotor activity, and increased susceptibility to CSD in heterozygous *Atp1a2* mice compared with the wild type. Learning and memory results are not consistent between the two heterozygous mouse studies. The Lingrel lab results showed poor learning and memory in the Morris water maze; however, the Kawakami lab found that the heterozygous mice had increased freezing during the conditioned fear stimuli which could be due to increased fear and anxiety, increased sensory perception, or improved learning and memory. Additional animal studies with the knock-in mutations associated with FHM2 and the *Atp1a2* knock-outs are needed to better understand FHM2, treatment options, and its overall effect on behavior.

## Abbreviations

Atp1a2: Na^+^/K^+^-ATPase α2; Atp1a2±: *Atp1a2* heterozygous; Ca2+: calcium; CNS: central nervous system; CSD: cortical spreading depression; FHM: familial hemiplegic migraine; FHM2: familial hemiplegic migraine type II; K+: potassium; Na+: sodium; TGVS: trigeminovascular system.

## Competing interests

The authors declare that they have no competing interests.

## Authors’ contributions

SMG drafted the manuscript. RAR helped draft the manuscript. All authors read and approved the final manuscript.
